# When treating canine diabetic ketoacidosis, do balanced crystalloids provide superior outcomes compared to 0.9% Saline?

**DOI:** 10.18849/ve.v7i3.599

**Published:** 2022-08-24

**Authors:** Sara Marella+

**Keywords:** BALANCED FLUID THERAPY, BUFFERED CRYSTALLOID, COMPLICATED DIABETES MELLITUS, DIABETIC EMERGENCIES, DIABETIC KETOACIDOSIS, FLUID THERAPY, NON-BUFFERED CRYSTALLOID FLUID, SALINE

## Abstract

PICO question

When treating canine diabetic ketoacidosis, do balanced crystalloids provide superior outcomes compared to 0.9% saline?

Clinical bottom line

Category of research question

Treatment and prognosis

The number and type of study designs reviewed

Zero

Strength of evidence

Zero

Outcomes reported

There is currently a lack of studies looking at comparing 0.9% saline to a buffered crystalloid solution (such as Hartmann's) in dogs with diabetic ketoacidosis

Conclusion

In view of the strength of evidence and the outcomes from the analysed studies, there is currently no evidence that the use of 0.9% saline or the use of a buffered crystalloid affects the outcome in dogs with diabetic ketoacidosis

How to apply this evidence in practice

The application of evidence into practice should take into account multiple factors, not limited to: individual clinical expertise, patient’s circumstances and owners’ values, country, location or clinic where you work, the individual case in front of you, the availability of therapies and resources.

Knowledge Summaries are a resource to help reinforce or inform decision making. They do not override the responsibility or judgement of the practitioner to do what is best for the animal in their care.

## Clinical scenario

A dog presents with a history of lethargy, polyuria, polydipsia, vomiting and anorexia. The physical examination is consistent with hypovolaemic shock. Initial diagnostics reveal hyperglycaemia, glycosuria, ketonaemia, a metabolic acidosis with high anion gap, hyponatraemia and hyperlactatemia.

This patient has diabetic ketoacidosis (DKA) and hypovolaemic shock. Is it better to use 0.9% saline or a balanced crystalloid solution as a replacement fluid therapy, and which fluid is better as maintenance fluid therapy? Should we give importance to the pH, to the dysnatraemia, to the risk of cerebral oedema or to the risk of hyperchloraemia?

## The evidence

No peer-reviewed scientific papers were identified that addressed the PICO question.

## Summary of the evidence

There was no peer-reviewed evidence that met the inclusion criteria to be summarised.

## Appraisal, application and reflection

Diabetic ketoacidosis (DKA) is one form of complicated diabetes mellitus. As these patients can have severe fluid losses, with electrolyte loss depending on the fluid composition, most of the patients with DKA show differing degrees of acid-base and electrolyte imbalances and alterations in effective osmolarity. As a consequence of these alterations, restoring an appropriate fluid balance, before starting insulin treatment, remains one of the cornerstones in the treatment of these patients (Boysen, 2008; Connally, 2002; Wolfsheimer, 1989; Chastain, 1981; and Hess, 2013). Electrolyte imbalances and severity of acidosis influences the prognosis in patients with DKA (Hess, 2013; Hume et al., 2006; Efstathiou et al., 2002; Ueda et al., 2015; Agarwal et al., 2016; and Ahuja et al., 2019).

A variety of crystalloid fluids are currently available but they differ in buffer composition and ion concentration. In selecting the most appropriate fluid therapy for DKA patients, there are two key elements to consider; sodium content in the fluid, and the alkalising / acidifying property of the fluid. Of course, these two characteristics need to be related to the patient’s serum osmolarity, serum sodium concentration and acid-base status.

In contrast to human medicine, where cerebral oedema is described as a complication of DKA, particularly in paediatric medicine, cerebral oedema is rarely a reported complication of DKA in veterinary medicine. Multiple mechanisms are responsible for its development, with the patients initial natraemia and osmolarity playing a key role, alongside the correction of these abnormalities associated with the pathogenesis of the condition (Boysen, 2008; and Long & Koyfman, 2017). One mechanism that results in alterations in sodium levels relates to the patients hyperglycaemic state; accumulation of glucose in the extracellular space leads to intra-cellular dehydration and dilutional hyponatraemia (pseudohyponatraemia). Secondary to the hyperglycaemic osmotic effect, it is sodium concentration (total body sodium content relative to extracellular water content) rather than total body sodium content that changes. Mathematical formulae have been extrapolated in order to estimate the natraemia (corrected sodium) in a normoglycaemic state, excluding the fluid shift caused by hyperglycaemia (Katz, 1973; and Hillier at al., 1999). However, other mechanisms, such as osmotic diuresis, excretion of ketoacids and gastro-intestinal losses, may result in total sodium depletion, all contributing to patient dysnatraemia (Connally, 2002; Wolfsheimer, 1989; Tabor, 2019; and Panciera, 2006).

Neurons are the most sensitive cells to alteration in osmolarity and in order to counter the extra osmolality of plasma during these episodes of hypertonicity, they accumulate intra-cytoplasmatic osmoles (Connally, 2002; Wolfsheimer, 1989; and Tabor, 2019).

The aim of initial fluid therapy in DKA is to improve hyperglycaemia along with patient natraemia without significant changes in osmolarity, or cerebral oedema and further neurologic dysfunction may occur (Connally, 2002; Wolfsheimer, 1989; Panciera, 2006; Nelson, 2015; Harris et al., 1990; Hoorn et al., 2007; Hale et al., 2008; Durward et al., 2011; and Schermerhorn & Barr, 2006).

This requires normal kidney function and a fluid therapy that aims to correct both the patient’s sodium and water deficits. Replacement fluid therapy reduces blood glucose and ketone concentrations through different mechanisms, such as dilution, increased glomerular filtration rate, decreased circulating catecholamines and reduction in antidiuretic hormone (ADH) and renin-angiotensin-aldosterone system (RAAS) activation.

In addition to dysnatraemia, DKA patients often have profound metabolic acidosis secondary to multiple mechanisms: overproduction of ketoacids, hypovolaemia (lactic acidosis, volume-responsive azotaemia) and hyperchloraemic metabolic acidosis. The latter occurs mainly as a result of chloride retention when keto-anions are excreted in the urine with sodium and potassium and / or secondary to administration of chloride-rich fluids (0.9% saline) (Boysen, 2008; Thomovsky, 2017; Semler et al., 2018; and Self et al., 2018). It is known that, due to its high chloride content, 0.9% saline can cause a non-anion gap metabolic acidosis with a reduction in serum strong ion difference; this acidosis is generally considered transient and of low impact (Wolfsheimer, 1989). Nevertheless, a causal link between hyperchloraemia and renal vasoconstriction has been hypothesised, which clinically may translate into a higher risk of acute kidney injury (Boysen, 2008; Williams et al., 2020; and Semler et al., 2018).

In comparison to chloride-rich saline, buffered crystalloids (such as Hartmann’s solution, Plasmalyte 148, Ringer’s acetate) have a more physiologic chloride concentration and contain anion buffers that avoids hyperchloraemic metabolic acidosis.  These balanced crystalloids are on average more expensive than 0.9% saline and some studies also argue that infusion of Ringer’s lactate solution may cause an elevation in serum lactate levels that may accumulate in cases of liver insufficiency, or may be converted into glucose, exacerbating hyperglycaemia (Boysen & Dorval, 2014). Finally, from experimental studies and theoretical conceptions it seems that the acetate present in Plasmalyte 148 and Ringer’s acetate may be associated with alterations in myocardial activity and patient hemodynamics (Boysen, 2008; Connally, 2002; and Ellekjaer et al., 2019).

From a pathophysiological point of view, it is evident that in DKA, the choice between 0.9% saline and buffered crystalloids is not straightforward; given their different compositions each fluid has advantages and disadvantages.

In veterinary medicine no clinical trials evaluate outcome for dogs with DKA that received 0.9% saline compared to a buffered crystalloid (such as Hartmann's). From the search strategy applied, no papers met the inclusion criteria for this Knowledge Summary.

Some veterinary opinion articles, not directly addressing the PICO question but reviewing the topic of fluid therapy in DKA, suggest 0.9% saline as the fluid of choice because this is the fluid recommended by most of the human DKA guidelines, the acidosis due to chloride-rich fluids is minimal and self-limiting, and its higher sodium content avoids rapid variation in osmolarity, reducing the risk of cerebral oedema. Moreover, the use of buffered crystalloids may lead to overshoot alkalosis when keto-anions and lactate are metabolised, negatively charged lactate can promote further sodium and potassium loss in the urine, and finally in the presence of severe dehydration or shock, a lactic acid may contribute to the already existing acidaemia (Boysen, 2008; Connally, 2002; Wolfsheimer, 1989; Hess, 2013; Nelson, 2000; Panciera, 2006; Nelson, 2015; and Macintire, 1995).

On the other hand, articles have questioned the use of 0.9% saline because of its acidifying effect and the lack of association between lower sodium concentrations in buffered crystalloids and a greater risk of cerebral oedema (Long & Koyfman, 2017). Finally, other veterinary opinion articles advise modification of the fluid therapy toward 0.9% saline or a balanced crystalloid on the basis of the acid-base and electrolyte status of the single patient, using a personalised approach in the choice of the fluid therapy (Wolfsheimer, 1989; Chastain, 1981; Gant, 2019; Tabor, 2019; Brown, 2009; Nelson, 2000; and Nelson, 2015).

Human medicine studies about development of cerebral oedema in DKA found that the type of fluid rarely plays a role; it seems that DKA cerebral oedema is mainly due to reperfusion of ischaemic brain tissue and increased vascular permeability, rather than shifting of water into brain cells, making fluid therapy rarely, if ever, a contributing factor (Chastain, 1981; Hume et al., 2006; Higgs, 2013; Gant, 2019; and Long & Koyfman, 2017).

In human medicine, the incidence of new or progressive acute kidney injury (AKI) and resolution of AKI was not different between patients in DKA that received 0.9% saline versus a balanced crystalloid (Williams et al., 2020). Furthermore, human medicine trials have compared outcomes in patients with DKA that received 0.9% saline versus a balanced crystalloid. No significant difference in outcomes (time to normalise pH or glycaemia, time to receive subcutaneous insulin, sodium alteration, duration of hospitalisation, resolution of DKA) has been found in most of the studies (Williams et al., 2020; Yung et al., 2017; Chua et al., 2012; Chiasson et al., 2003; Van Zyl et al., 2012; Fayfman et al., 2017; and Self et al., 2020).Whilst this was not a primary outcome, one controlled trial found that patients that received Hartmann’s solution, in comparison to 0.9% saline, had a statistically significant shorter length of hospitalisation (Yung et al., 2017). A secondary analysis of two cluster randomised clinical trials found that treatment with balanced crystalloids resulted in more rapid resolution of DKA, compared with 0.9% saline (Self et al., 2020).

Based on several recent human randomised clinical trials comparing buffered crystalloids vs 0.9% saline, buffered solutions are increasingly recommended as first-line replacement fluid. Despite this, most of the DKA human guidelines suggest the use of 0.9% saline as the first fluid of choice (Jayashree et al., 2019; Tran et al., 2017; Fayfman et al., 2017; and NICE, 2015).

As already mentioned, there is currently no evidence in veterinary medicine if any particular type of crystalloid fluid is superior to another in hyperglycaemic emergencies. However, veterinary opinion articles and human studies represent a starting point in the analysis of which type of fluid therapy is better in a patient with DKA, highlighting the importance in evaluating certain elements of a fluid’s composition (sodium content and alkalising / acidifying property) that may change the outcome in patients with DKA and also may shorten hospitalisation time, potentially reducing the cost of treatment and mortality associated with euthanasia.

In conclusion, there is zero evidence if one particular isotonic crystalloid fluid confers a clinical outcome benefit over others in the treatment of DKA in veterinary medicine. A personalised fluid therapy plan that considers the patient’s specific acid-base balance and electrolytes and re-evaluates these values during the treatment may be the best solution in this complex pathology.

## Methodology Section

**Search Strategy:** 

Databases searched and dates covered:	CAB Abstracts on OVID Platform–1973 to 2022 Week 08 PubMed accessed via the NCBI website 1920–February 2022 Hand search
Search strategy:	CAB Abstracts: (dog or dogs or canine or canines).mp. or exp dogs/ (diabetic or diabetes).mp. or exp diabetes/ (DKA or ketoacid* or acidosis or complicat* or emergenc*).mp. (saline or 'sodium chloride' or NaCl or 'non buffered crystalloid').mp. ('buffered crystalloid' or 'balanced crystalloid' or 'balanced fluid' or Hartmann* or Ringer* or 'sodium lactate' or isotonic or 'balanced salt' or plasma-lyte or plasma lyte or normosol or 'fluid therapy') 1 and 2 and 3 and (4 or 5) PubMed: dog or dogs or canine or canines diabetic or diabetes DKA or ketoacid or acidosis or complication or emergency saline or 'sodium chloride' or NaCl or 'non buffered crystalloid' 'buffered crystalloid' or 'balanced crystalloid' or 'balanced fluid' or Hartmanns or Ringers or 'sodium lactate' or isotonic or 'balanced salt' or plasma-lyte or plasma lyte or normosol or 'fluid therapy' 1 and 2 and 3 and (4 or 5)
Dates searches performed:	01 Mar 2022

**Exclusion / Inclusion Criteria:** 

Exclusion:	Not relevant to PICO. No full text available. Non-English language publications. Duplicate papers. Conference proceedings. Articles involving the wrong species.
Inclusion:	Article relevant to the PICO question. Full text available. Papers relevant to veterinary medicine.

**Search Outcome:** 

Database	Number of results	Excluded – Not relevant to PICO	Excluded – Inaccessible	Excluded – Non-English language publications	Excluded – Duplicates	Excluded – Conference proceedings	Total relevant papers
CAB Abstracts	56	27	2	7	0	20	0
PubMed	60	60	0	0	0	0	0
Hand Search	4	4	0	0	0	0	0
Total relevant papers when duplicates removed							0

## Conflict of interest

The authors declare no conflicts of interest.
